# Supplementary material for: Spatiotemporal evaporating droplet dynamics on fomites enhances long term bacterial pathogenesis
Source: Commun Biol. 2021 Oct 8;4:1173. doi: 10.1038/s42003-021-02711-z (PMC8501104; doi:10.1038/s42003-021-02711-z)
Supplement: Supplementary file 2 — Supplementary Information [file 42003_2021_2711_MOESM2_ESM.pdf]

Supplementary Materials for

Spatiotemporal evaporating droplet dynamics on fomites enhances long term bacterial pathogenesis

Sreeparna Majee[1], Atish Roy Chowdhury[2], Roven Pinto[1+], Ankur Chattopadhyay[1+], Amey Nitin Agharkar[3], Dipshikha Chakravortty[2*], Saptarshi Basu[1,3**]

**To whom correspondence should be addressed; E-mail: sbasu@iisc.ac.in.
*Co-Corresponding author; E-mail: dipa@iisc.ac.in.
+These authors contributed equally to the work.

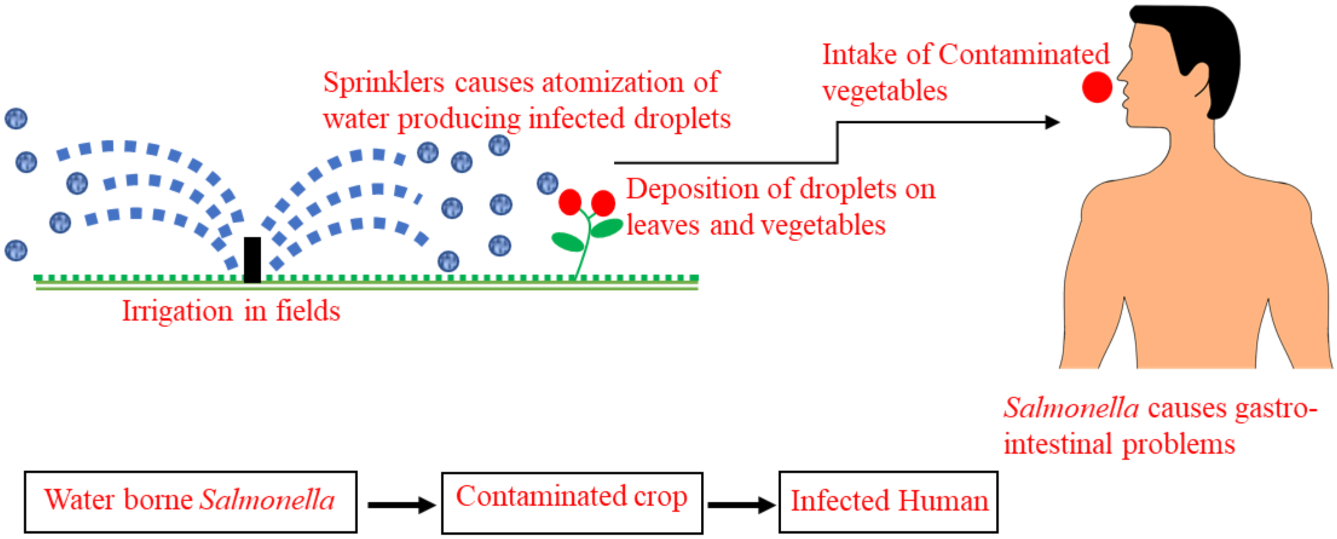

Supplementary Figure 1.
Schematic diagram. Contamination through droplets produced during irrigation.

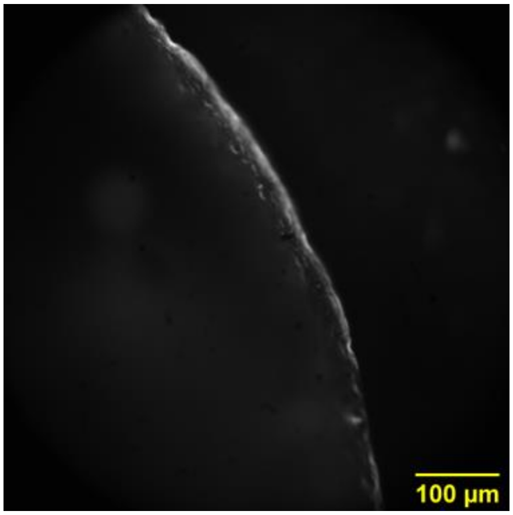

Supplementary Figure 2.

Fluorescence image (measured at 50X): Dried precipitate of 0.6 wt% mucin containing STM (WT) on tomato skin.

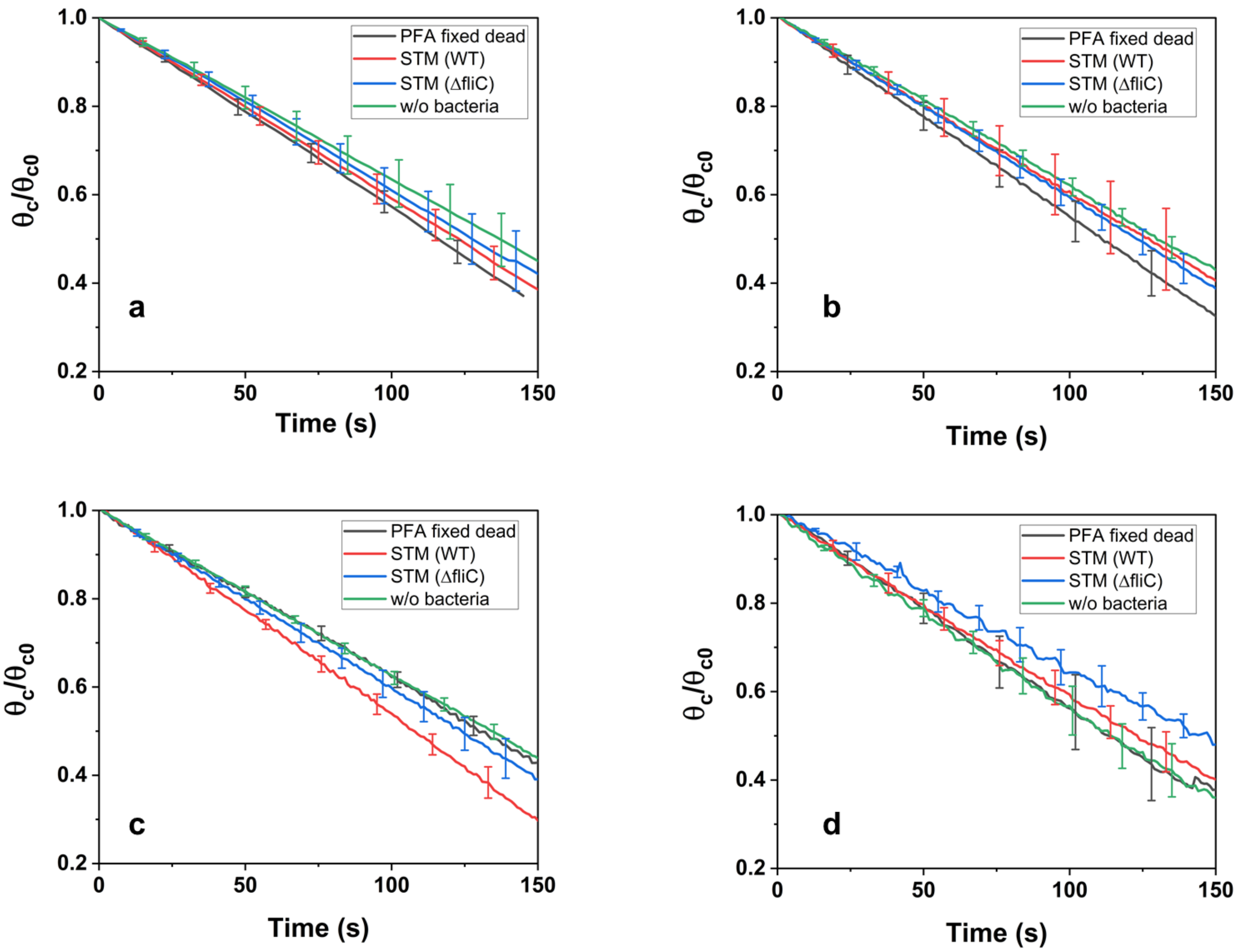

Supplementary Figure 3.

Contact angle vs. time. Variation in normalized contact angle with time for four mediums (a) Saline (0.9wt%), (b) Milli-Q, (c) Dextrose (5wt%), (d) Mucin (0.6wt%)

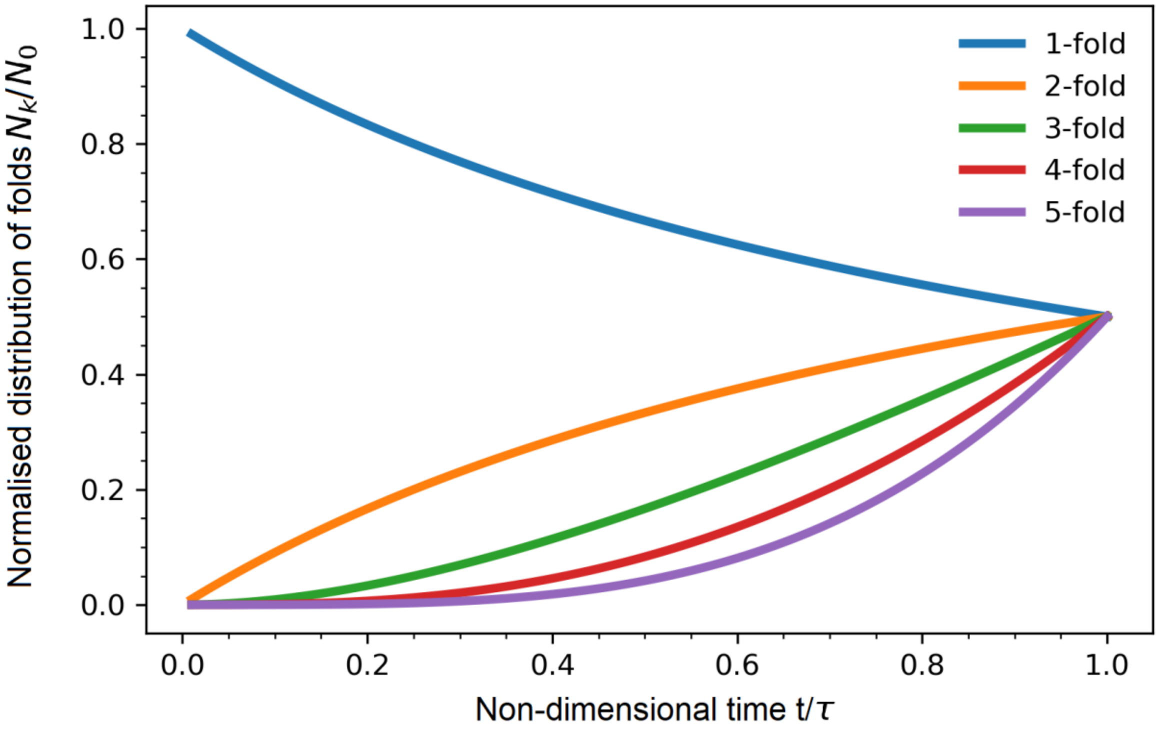

Supplementary Figure 4.

Normalized distribution of folds with time. Fold generation of bacteria (1-5 folds) given in Model B in the control volume mentioned in the main text. The time is normalized with τ which is the characteristic time of aggregation.

.

Supplementary Table 1.
Viscosity of different mediums in mPa.s.

**Fluid Mediums (w/o bacteria)**	**Viscosity (mPa.s)**
**Milli-Q**	**1.1**
**Saline (0.9wt%)**	**1.2**
**Dextrose (5wt%)**	**1.3**
**Dextrose (0.9wt%)**	**1.2**
**Mucin (0.6wt%)**	**1.7**
**Mucin (0.1wt%)**	**1.3**

Supplementary Table 2.
Surface tension of different mediums in mN/m.

**Fluid Medium**	**w/o bacteria**	**PFA fixed dead**	**STM (WT)**	**STM (Δ*fliC*)**
**Saline (0.9wt%)**	**70.9 ± 2.7**	**70.7 ± 3.2**	**70.4 ± 3.1**	**70.6 ± 1.8**
**Milli-Q**	**70.7 ± 1.8**	**71.7 ± 2.7**	**70.4 ± 1.8**	**70.8 ± 2.5**
**Dextrose (5wt%)**	**71.6 ± 1.9**	**70.8 ± 1.9**	**71.3 ± 2.4**	**71.7 ± 3.3**
**Mucin (0.6wt%)**	**71.8 ± 2.8**	**70.6 ± 2.7**	**70.4 ± 3.2**	**70.7 ± 2.8**
