# Supplementary material for: Spatiotemporal evaporating droplet dynamics on fomites enhances long term bacterial pathogenesis
Source: Commun Biol. 2021 Oct 8;4:1173. doi: 10.1038/s42003-021-02711-z (PMC8501104; doi:10.1038/s42003-021-02711-z)
Supplement: Supplementary file 3 — Description of Supplementary Files [file 42003_2021_2711_MOESM3_ESM.pdf]

Description of Additional Supplementary Files

File name: Supplementary Movie 1.
Description: Capillary Flow. Bacteria showing capillary flow (Saline (0.9wt%) ΔfliC).

File name: Supplementary Movie 2.
Description: Live cell imaging of Salmonella Typhimurium towards the edge of the droplet in Dextrose (5wt\%) medium. The edge of the droplet is imaged under 63x magnification by Leica SP8 confocal microscope focusing on the flow dynamics in dextrose medium.

File name: Supplementary Movie 3.
Description: Live cell imaging of Salmonella Typhimurium towards the edge of the droplet in Mucin (0.6wt\%) medium. The edge of the droplet is imaged under 63x magnification by Leica SP8 confocal microscope focusing on the flow dynamics in mucin medium.

File name: Supplementary Movie 4.
Description: Comparison between dead and STM WT bacteria flowing towards the edge of the droplet in Saline (0.9wt\%) medium. Simultaneous live cell imaging of droplet edge with dead and STM WT bacteria in saline solution captured under 63x magnification by Leica SP8 confocal microscope.

File name: Supplementary Data 1.
Description: Data for fluorescence intensity at the edge for dead bacteria, STM (WT) and STM (Δ*fliC)* in saline medium.

File name: Supplementary Data 2.
Description: Data for fluorescence intensity at the edge for dead bacteria, STM (WT) and STM (Δ*fliC)* in milli-Q medium.

File name: Supplementary Data 3.
Description: Data for radial profilometry for dead bacteria, STM (WT) and STM (Δ*fliC)* in dextrose.

File name: Supplementary Data 4.
Description: Data for radial profilometry for dead bacteria, STM (WT) and STM ((Δ*fliC)* in mucin.

File name: Supplementary Data 5.
Description: Data for velocity profiles for saline and Page 8 of 18 nutrient rich media at the later stages of evaporation.

File name: Supplementary Data 6.
Description: Data for normalized contact angle variation with time for saline, mucin and dextrose.

File name: Supplementary Data 7.
Description: Data for the viability of wild type and fliC deficient strains of Salmonella.

File name: Supplementary Data 8.
Description: Data for viable Salmonella Typhimurium present in fomite can cause infection in mouse model.

File name: Supplementary Data 9.
Description: Data for variation in normalized contact angle with time for Saline (0.9wt%).

File name: Supplementary Data 10.
Description: Data for variation in normalized contact angle with time for Milli-Q.

File name: Supplementary Data 11.
Description: Data for variation in normalized contact angle with time for Dextrose (5wt%).

File name: Supplementary Data 12.
Description: Data for variation in normalized contact angle with time for Mucin (0.6wt%).
