# Supplementary material for: Spatiotemporal evaporating droplet dynamics on fomites enhances long term bacterial pathogenesis
Source: Commun Biol. 2021 Oct 8;4:1173. doi: 10.1038/s42003-021-02711-z (PMC8501104; doi:10.1038/s42003-021-02711-z)
Supplement: Supplementary file 20 — Reporting summary [file 42003_2021_2711_MOESM20_ESM.pdf]

nature portfolio

Corresponding author(s): Prof. Saptarshi Basu
Prof. Dipshikha Chakravortty

Last updated by author(s): Sep 17, 2021

Reporting Summary

Nature Portfolio wishes to improve the reproducibility of the work that we publish. This form provides structure for consistency and transparency in reporting. For further information on Nature Portfolio policies, see our Editorial Policies and the Editorial Policy Checklist.

Statistics

For all statistical analyses, confirm that the following items are present in the figure legend, table legend, main text, or Methods section.

n/a	Confirmed	
☐	☒	The exact sample size (*n*) for each experimental group/condition, given as a discrete number and unit of measurement
☐	☒	A statement on whether measurements were taken from distinct samples or whether the same sample was measured repeatedly
☐	☒	The statistical test(s) used AND whether they are one- or two-sided<br>*Only common tests should be described solely by name; describe more complex techniques in the Methods section.*
☐	☒	A description of all covariates tested
☐	☒	A description of any assumptions or corrections, such as tests of normality and adjustment for multiple comparisons
☒	☐	A full description of the statistical parameters including central tendency (e.g. means) or other basic estimates (e.g. regression coefficient) AND variation (e.g. standard deviation) or associated estimates of uncertainty (e.g. confidence intervals)
☐	☒	For null hypothesis testing, the test statistic (e.g. *F*, *t*, *r*) with confidence intervals, effect sizes, degrees of freedom and *P* value noted<br>*Give P values as exact values whenever suitable.*
☒	☐	For Bayesian analysis, information on the choice of priors and Markov chain Monte Carlo settings
☒	☐	For hierarchical and complex designs, identification of the appropriate level for tests and full reporting of outcomes
☒	☐	Estimates of effect sizes (e.g. Cohen's *d*, Pearson's *r*), indicating how they were calculated

Our web collection on statistics for biologists contains articles on many of the points above.

Software and code

Policy information about availability of computer code

Data collection	Custom made code in MATLAB R2020a and PYTHON 3.8
Data analysis	MS Excel 2016, LaVisionDaVis 8.4, Origin Pro 2021 (Learning Edition), ImageJ, GraphPad Prism 8, Zen Black (Carl Zeiss), LasX (Leica)

For manuscripts utilizing custom algorithms or software that are central to the research but not yet described in published literature, software must be made available to editors and reviewers. We strongly encourage code deposition in a community repository (e.g. GitHub). See the Nature Portfolio guidelines for submitting code & software for further information.

Data

Policy information about availability of data

All manuscripts must include a data availability statement. This statement should provide the following information, where applicable:

- Accession codes, unique identifiers, or web links for publicly available datasets
- A description of any restrictions on data availability
- For clinical datasets or third party data, please ensure that the statement adheres to our policy

All data are available in the main text and supplementary materials. All material and codes are available from the corresponding author upon request.

Field-specific reporting

Life sciences study design

All studies must disclose on these points even when the disclosure is negative.

Sample size	All the cell line infection studies were done twice independently (biological replicates) with at least four technical replicates. The intra-macrophage survival of the wild type bacteria collected from different surface (door handle, tomato, cucumber etc) was tested once with four technical replicates. The confocal imaging experiment was performed twice with ten technical replicates. In the study of measuring of bacterial viability in vitro, conducted in saline and Milli Q water, three biological replicates were present. In the time dependent viability measurement study conducted in high and low concentrations of mucin and dextrose droplets , three independent experiments were performed. The animal experiments have been performed once with three mice in each group. All the sample sizes have been mentioned in the figure legends. 'n' denotes the technical replicates, where as 'N' has been used to demonstrate biological replicates.
Data exclusions	For the exclusion of any data, out lier tests have been performed when the graphs were being plotted using graphPad Prism 8 software. Mostly unpaired student's t test and 2 way ANOVA were used for statistical analysis. The name of the statistical tests used in the study have been mentioned in the figure legends. The high reproducibility of the data reduced any further scope of data exclusion.
Replication	Most of the biological experiments are performed twice independently with multiple technical replicates. The experimental data were reproducible. In the animal experiments three 4 to 6 weeks old healthy mice were kept in each cohort. The data represented in the in vitro viability study was also reproducible.
Randomization	The allocation of the samples/bacterial cells/eukaryotic cells were completely random.
Blinding	Investigators were blinded to group allocation during data collection and analysis of the data.

Reporting for specific materials, systems and methods

We require information from authors about some types of materials, experimental systems and methods used in many studies. Here, indicate whether each material, system or method listed is relevant to your study. If you are not sure if a list item applies to your research, read the appropriate section before selecting a response.

Materials & experimental systems

n/a	Involved in the study	
☐	☒	Antibodies
☐	☒	Eukaryotic cell lines
☒	☐	Palaeontology and archaeology
☐	☒	Animals and other organisms
☒	☐	Human research participants
☒	☐	Clinical data
☒	☐	Dual use research of concern

Methods

n/a	Involved in the study	
☒	☐	ChIP-seq
☒	☐	Flow cytometry
☒	☐	MRI-based neuroimaging

Antibodies

Antibodies used	Anti mouse LAMP-1 antibody, host-rat, manufacturer- DSHB, antibody registry id-AB_528127<br>Anti rat dylight 488 secondary antibody, host-goat, Jackson's Immuno Research Laboratories, AB_2338327<br>Anti Salmonella antibody, host-rabbit, specific for o antigen of bacterial outer membrane, source- lab stock<br>Anti rabbit cy3 secondary antibody, host-goat, Jackson's Immuno Research Laboratory, AB_2338011
Validation	Anti mouse LAMP-1 antibody, host-rat, manufacturer- DSHB, antibody registry id-AB_528127<br>Anti Salmonella antibody, host-rabbit, specific for o antigen of bacterial outer membrane, source- lab stock

Eukaryotic cell lines

Policy information about cell lines

Cell line source(s)	RAW 264.7 cells from laboratory stocks.
Authentication	Morphological examination by microscopy.
Mycoplasma contamination	The cells were maintained in sterile condition with 1X concentration of penicillin and streptomycin to remove mycoplasma contamination.
Commonly misidentified lines (See ICLAC register)	NA.

Animals and other organisms

Policy information about studies involving animals; ARRIVE guidelines recommended for reporting animal research

Laboratory animals	BALB/c mice, 4-6 weeks old male.
Wild animals	NA.
Field-collected samples	NA.
Ethics oversight	The animal experiments were sanctioned by the Institutional Animal Ethics Committee and the guidelines obtained from the National Animal Care were followed. (Registration number: 48/1999/CPCSEA).

Note that full information on the approval of the study protocol must also be provided in the manuscript.
